# Objective Macular Asymmetry Metrics for Glaucoma Detection Using a Temporal Raphe–Based OCT Linearization Algorithm

**DOI:** 10.3390/jcm15020461

**Published:** 2026-01-07

**Authors:** Takuhei Shoji, Miho Seo, Hisashi Ibuki, Hirokazu Ishii, Junji Kanno, Kei Shinoda

**Affiliations:** 1 Department of Ophthalmology, Saitama Medical University, 38 Morohongo Moroyama-Machi, Iruma 350-0495, Saitama, Japanshinodak@med.teikyo-u.ac.jp (K.S.); 2 Koedo Eye Institute, Kawagoe 350-1123, Saitama, Japan

**Keywords:** glaucoma, optical coherence tomography, macular asymmetry, temporal raphe, diagnosis, ganglion cell complex

## Abstract

**Highlights:**

**What is already known:**

Macular inner retinal layer asymmetry is associated with glaucoma, but objective and standardized quantification has been limited.

**What this study adds:**

A fully automated linearization program was developed to align the disc–fovea and temporal raphe axes, enabling reliable macular asymmetry scoring with high diagnostic performance.

**How this might affect practice:**

Objective asymmetry metrics may help identify unilateral or asymmetric glaucomatous changes and support standardized macular OCT interpretation; external validation in broader and bilateral-disease cohorts is required.

**Abstract:**

**Background/Objectives:** We aim to develop an image linearization process and a program capable of quantifying vertical and left–right asymmetries observed in macular scans. We then sought to verify its applicability in clinical settings. **Methods:** In this single-center cross-sectional study, we examined 37 consecutive patients with unilateral open-angle glaucoma and analyzed paired data (glaucomatous eye vs. fellow normal eye). Spectral-domain OCT images were automatically processed by a custom program to align the disc–fovea axis and temporal raphe, and the following parameters were evaluated: (1) mean inner retinal thickness difference (superior–inferior), (2) Vertical Asymmetry Score, and (3) Quadrantal Asymmetry Score. **Results:** We analyzed 37 healthy eyes and 37 POAG eyes. After linearization, the mean inner retinal thicknesses for the normal and POAG groups were 93.4 µm (interquartile range [IQR]: 90.1–98.5) and 80.3 µm (IQR: 77.3–85.0), respectively. The Vertical Asymmetry Score was 6.80 (IQR: 6.15–7.25) for healthy eyes and 9.69 (IQR: 9.16–11.58) for POAG eyes. The Quadrantal Asymmetry Score was 6.35 (IQR: 5.94–7.19) for healthy eyes and 8.47 (IQR: 8.11–9.63) for POAG eyes. Significant differences were found between groups for all parameters (*p* < 0.001). The Vertical Asymmetry Score (AUC = 0.967, *p* < 0.001) and Quadrantal Asymmetry Score (AUC = 0.946, *p* < 0.001) demonstrated significantly greater accuracy in detecting glaucoma compared to the mean inner retinal thickness (AUC = 0.743). **Conclusions:** The developed linearization program and asymmetry scores have shown promise as parameters for objectively quantifying macular asymmetry using spectral-domain OCT. External validation in independent cohorts, including bilateral disease, is warranted.

## 1. Introduction

Glaucoma, a chronic and irreversible ocular condition, is distinguished by the degeneration and depletion of retinal ganglion cells (RGCs) and their corresponding axons within the retina [1,2,3,4]. This progressive optic-nerve impairment consequently leads to the manifestation of distinct visual-field abnormalities. The deleterious impact of glaucoma on visual health is of significant concern, as it stands as the second most prevalent cause of avoidable blindness globally [5]. According to recent statistics, an estimated 3.6 million individuals aged 50 years and above experienced vision loss due to glaucoma in the year 2020 [6]. This alarming figure highlights the urgent need for enhanced preventive measures, early detection strategies, and effective therapeutic interventions to mitigate the growing burden of glaucoma-related visual impairment.

Optical Coherence Tomography (OCT), a non-invasive imaging technique, has emerged as a valuable tool in diagnosing and monitoring glaucoma by providing detailed structural information of the retina and optic nerve head. In recent years, researchers have turned their attention to exploring novel OCT biomarkers that may aid in the early detection and characterization of glaucoma. The temporal raphe, a distinctive anatomical landmark in the retina, has garnered considerable interest as a potential biomarker in glaucoma research [7,8,9,10,11,12,13,14,15]. It delineates the horizontal demarcation between the superior and inferior hemispheres of the retina, and its integrity assumes a pivotal role in sustaining retinal functionality. Recent investigations employing OCT imaging have identified diverse temporal raphe indications, encompassing thinning, which hold potential as markers for the recognition of glaucomatous retinal neurodegeneration and early-stage glaucomatous impairment. Nevertheless, the assessment of such vertical asymmetry within the macula presently remains a subjective appraisal reliant on the observer, lacking instruments equipped with integrated analysis programs for its quantitative evaluation.

Thus, the principal objective of this study is to develop a program capable of quantifying the vertical asymmetry, left–right asymmetry, or both, observed in macular scans, and to subsequently verify its applicability in clinical settings.

## 2. Methods

### 2.1. Study Enrollment and Participants

This cross-sectional study was approved by the Ethics Committee of Saitama Medical University (No. byou2023-051; 9 August 2023) and was conducted in accordance with the Declaration of Helsinki. Owing to the retrospective nature of this study, the requirement for additional informed consent was waived.

All participants underwent a comprehensive ophthalmologic examination, including best-corrected visual acuity (BCVA), slit-lamp biomicroscopy, intraocular pressure (IOP) measurement using Goldmann applanation tonometry, and dilated fundus examination. Standard automated perimetry (SAP) was performed using the Humphrey Field Analyzer (Carl Zeiss Meditec, Dublin, CA, USA) with the 24-2 Swedish Interactive Threshold Algorithm (SITA) Standard program. Axial length (AL) and central corneal thickness (CCT) were measured using an optical biometer (OA-2000, Tomey Corp., Nagoya, Japan). Eligible eyes were required to have BCVA of 20/50 or better, AL < 30 mm, reliable SAP results (<20% fixation loss, <15% false-negative errors, and <15% false-positive errors), and adequate OCT image quality (signal strength > 7). No exclusions were applied for refractive error, CCT, or prior ocular surgery if the above conditions were met.

Eyes were excluded if they had a history of glaucoma surgery, vitreoretinal or corneal surgery, clinically significant cataract, corneal opacity, retinal disease, or non-glaucomatous optic neuropathy. Eyes with pathological myopia or suspected pathologic changes, including diffuse or patchy chorioretinal atrophy, lacquer cracks, myopic choroidal neovascularization, or macular atrophy, were excluded to avoid confounding glaucomatous structural alterations.

### 2.2. Glaucoma Diagnosis

Glaucoma was diagnosed based on characteristic optic nerve head (ONH) changes and corresponding visual field defects. ONH abnormalities included a vertical cup-to-disc ratio ≥ 0.7, neuroretinal rim narrowing or notching (rim width ≤ 0.1), and/or localized retinal nerve fiber layer (RNFL) defects whose edges at the ONH margin exceeded those at adjacent major retinal vessels and diverged in arcuate or wedge-shaped patterns. Visual field defects were deemed glaucomatous when at least one of the Anderson–Patella criteria was satisfied: (1) a cluster of ≥3 points in the pattern deviation plot within one hemifield (superior or inferior) with *p* < 0.05, including ≥1 point at *p* < 0.01; (2) a glaucoma hemifield test result outside normal limits; or (3) pattern standard deviation with *p* < 0.05. Glaucomatous eyes were required to have open angles on gonioscopy. Pre-perimetric glaucomatous eyes presenting ONH structural changes without VF defects were also included. The study population primarily consisted of early-stage glaucoma cases, although patients with moderate to advanced stages were also included. The detailed demographic and clinical characteristics, including the range of visual field defects, are presented in Table 1. Diagnosis for each eye was made independently by two glaucoma specialists (M.S. and H.I.), with disagreements resolved by a third adjudicator (T.S.). Normal fellow eyes were required to have IOP < 21 mmHg, normal-appearing ONH and RNFL on fundus examination and stereo disc photographs, and no VF abnormalities.

### 2.3. Canon OCT Imaging

All participants underwent macular imaging using spectral-domain OCT (Xephilio OCT-A1. Canon Inc., Tokyo, Japan). The system operates at a wavelength of 855 nm with an A-scan rate of 70,000 scans per second, providing axial and transverse resolutions of approximately 3 µm and 20 µm, respectively. OCT images were acquired using the ‘Both Eyes’ macular scanning mode. To evaluate macular asymmetry, we utilized a non-commercial, internal research software provided by Canon Inc. This program automatically detects the foveal center and the optic disc center from the OCT volume data to establish the disc–fovea axis and execute the linearization algorithm. Because the processing is fully automated and deterministic, the computational reproducibility is 100% for the same image dataset. 

Macular inner retinal layer thickness measurements were automatically obtained for subsequent analysis.

### 2.4. Image Processing

**Figure 1:**
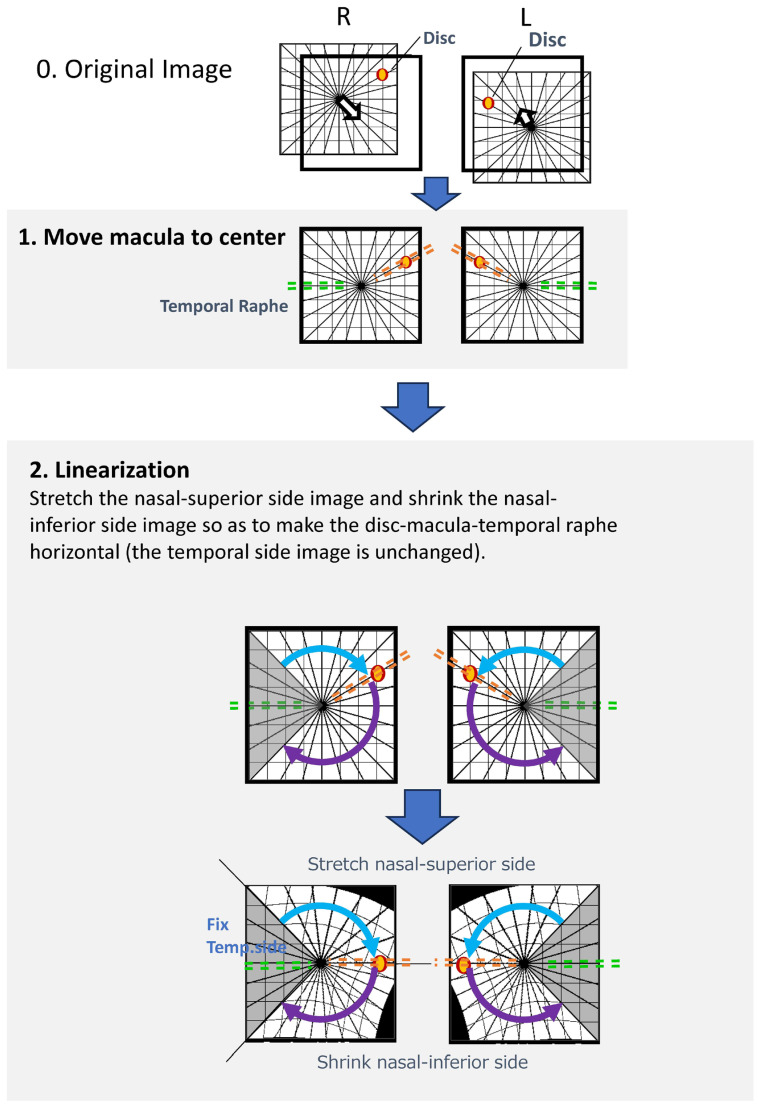
Image Processing Method; The custom image-processing pipeline consists of the following steps: 1. Foveal Centering: A built-in algorithm automatically detects the foveal center and positions it at the center of the OCT image. 2. Disc–Fovea Alignment: The center of the optic disc is automatically identified, and the image is rotated so that the disc–fovea axis becomes horizontal. The resulting image is referred to as the “*Rotation image*”; 3. Linearization Procedure: The region above the disc–fovea line is stretched.; The region below the disc–fovea line is compressed.; The temporal region within ±60 degrees of the fovea is preserved without distortion.; This transformation aligns both the disc–fovea line and the temporal raphe horizontally, enabling standardized geometric comparison of corresponding macular locations.

Image processing was performed using a custom-developed algorithm (Figure 1). The specific processing steps were as follows:

1. Foveal Detection: A built-in algorithm automatically detected the foveal center and positioned it at the center of the OCT image.
2. Optic Disc Detection and Rotation: The center of the optic disc was identified based on the geometric center of the low-reflectivity area corresponding to the neural canal opening. Each image was then rotated such that the disc–fovea axis became horizontal (referred to as the “Rotation image”).
3. Linearization Program: An affine transformation was applied to align the anatomical structures. After detecting the disc and foveal centers, the region superior to the disc–fovea line was vertically stretched, and the region inferior to it was compressed to ensure complete horizontal alignment of the disc–fovea axis. The temporal region within ±60 degrees from the fovea was preserved without distortion to maintain the anatomical configuration of the temporal raphe.
4. Thickness Computation: Retinal thickness at corresponding locations after linearization was computed using a nearest-neighbor matching algorithm, a widely used method for point-to-point correspondence following geometric transformation.

This procedure enabled standardized comparison of symmetric points across the macula. In the current study, the algorithm demonstrated robust performance, successfully processing all 37 scans (100% success rate) without any exclusions due to segmentation or registration failure.

### 2.5. Calculation of Asymmetry Metrics

To evaluate macular asymmetry, we employed two distinct approaches: a standard arithmetic difference for the conventional metric and a logarithmic ratio-based approach for the novel asymmetry scores.

#### 2.5.1. Mean GCC Thickness Difference (Conventional Metric)

As a reference for standard clinical assessment, the Mean GCC Thickness Difference was calculated as the absolute arithmetic difference in mean GCC thickness between the superior and inferior hemispheres.

#### 2.5.2. Vertical Asymmetry Score

**Figure 2:**
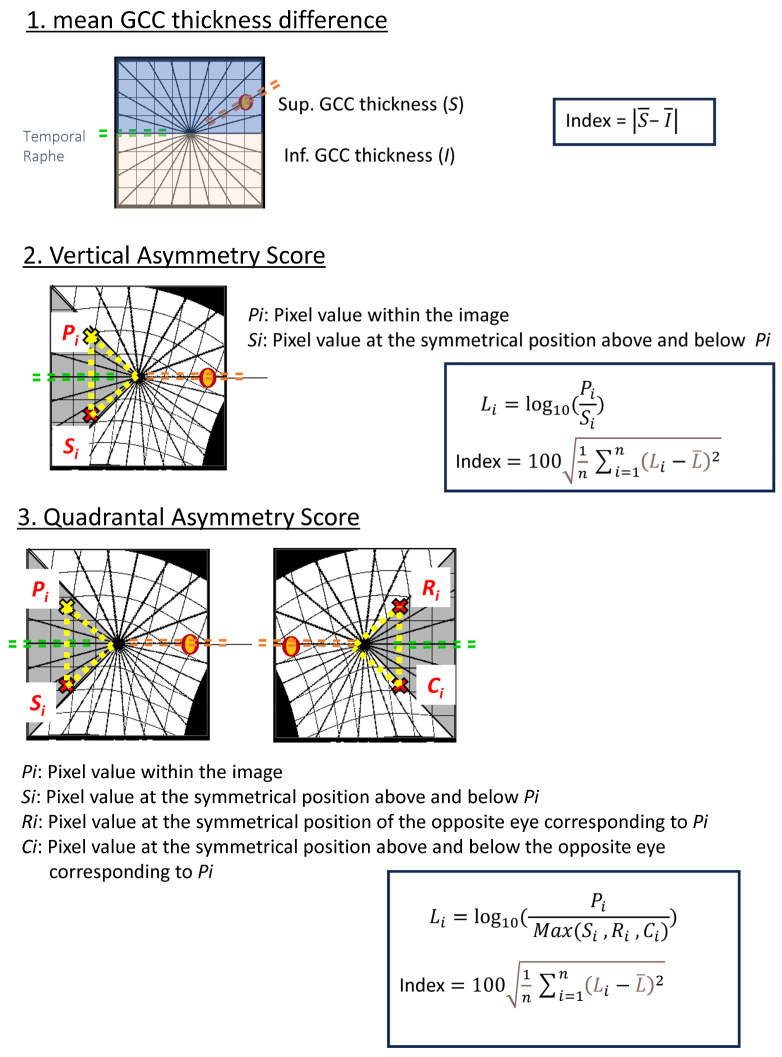
Calculation Methods for Each Parameter. (1) Mean GCC Thickness Difference: Pointwise differences in ganglion cell complex (GCC) thickness are computed across the macula. (2) Vertical Asymmetry Score: For each macular pixel $P_{i}$, the GCC thickness difference from its vertically mirrored counterpart $S_{i}$ is calculated. The standard deviation of all pointwise differences, multiplied by 100, defines the Vertical Asymmetry Score. (3) Quadrantal Asymmetry Score: For each pixel $P_{i}$, GCC thickness is compared with three symmetric locations: vertically mirrored $S_{i}$, horizontally/temporal raphe–mirrored $R_{i}$, and the corresponding mirrored point in the fellow eye $C_{i}$. The maximum of these symmetric reference values is identified, and the difference from $P_{i}$ is calculated. The standard deviation of these differences, multiplied by 100, defines the Quadrantal Asymmetry Score. Diagrammatic representations show pixel correspondences used for each parameter.

For the novel asymmetry scoring, we utilized a log-ratio approach to capture relative local variations. For each pixel Pi within the image, the logarithmic ratio of GCC thickness relative to its vertically symmetric point *S_i_* (above and below the fovea) was calculated (*L_i_* = log10(*P_i_*/*S_i_*)). The standard deviation of these pointwise log-ratios across the entire image (up to 65,536 points), multiplied by 100, was defined as the Vertical Asymmetry Score (Figure 2). 

#### 2.5.3. Quadrantal Asymmetry Score

Similarly, for the Quadrantal Asymmetry Score, the GCC thickness at each measurement point Pi was compared with three corresponding symmetric points: vertically mirrored (*S_i_*), horizontally mirrored (*R_i_*), and the corresponding point in the fellow eye (*C_i_*). The logarithmic ratio was calculated between *P_i_* and the maximum GCC thickness among these three reference points (*L_i_* = log10(*P_i_*/Max(*S_i_*, *R_i_*, *C_i_*))). The standard deviation of all resulting log-ratios, multiplied by 100, was defined as the Quadrantal Asymmetry Score.

To calculate the inter-eye asymmetry, data from left eyes were horizontally flipped (mirrored) to correspond to the right eye orientation. The superior-temporal quadrant of the left eye was thus compared to the superior-temporal quadrant of the right eye.

### 2.6. Statistical Analysis

Statistical analyses were performed using JMP Pro version 16 (SAS Institute, Cary, NC, USA). The normality of the data distribution was assessed using the Shapiro–Wilk test. Continuous variables are presented as mean ± standard deviation (SD) for normally distributed data and as median (interquartile range [IQR]) for non-normally distributed data. Since this study compared glaucomatous eyes with their fellow normal eyes, paired statistical tests were employed. Differences between the two groups were analyzed using the paired *t*-test for normally distributed variables and the Wilcoxon signed-rank test for non-normally distributed variables. Receiver operating characteristic (ROC) curves were generated to evaluate the diagnostic performance of each parameter. The area under the ROC curve (AUC) was calculated and compared between parameters. Comparison of AUCs between different metrics was performed using the roccomp command in Stata version 16.0 (StataCorp LLC, College Station, TX, USA), which implements the method of DeLong et al. to test the statistical significance of the difference between the areas under dependent ROC curves.

A post hoc power analysis indicated that the sample size of 37 pairs provided a power of >0.9 to detect significant differences in the asymmetry scores between groups, given the observed effect sizes.

## 3. Results

### 3.1. Participant Characteristics

A total of 37 patients with unilateral glaucoma were included (37 glaucomatous eyes and 37 fellow normal eyes). The median age of the participants was 68 years. There were no significant differences between glaucomatous and normal eyes in visual acuity, central corneal thickness, intraocular pressure, or axial length. As expected, visual field sensitivity differed significantly between the two groups. The demographic and clinical characteristics are summarized in Table 1.

### 3.2. OCT Parameters

**Figure 3:**
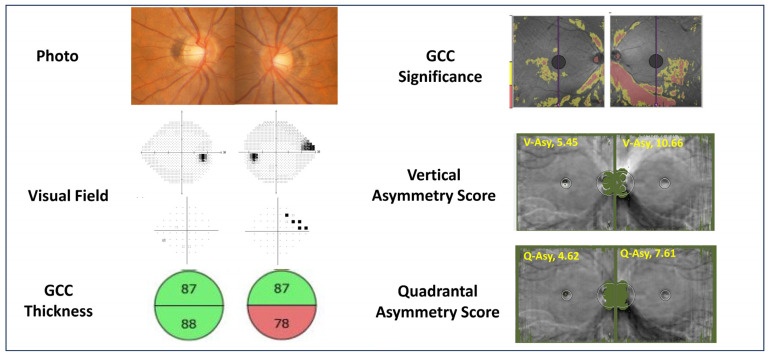
A representative case of a 48-year-old woman with unilateral glaucoma. The **left panels** show the color fundus photograph, visual field test results, macular ganglion cell complex (GCC) thickness map, and GCC significance map. The **right panels** display the corresponding asymmetry analyses obtained using the linearization program. In the visual field panels, the black areas in the grayscale map indicate reduced sensitivity (visual field defects). The black squares in the pattern deviation plot represent statistically significant sensitivity loss (*p* < 5% to *p* < 0.5%). In the GCC significance map, the colors represent the deviation from the normative database: green indicates normal thickness, and red indicates significant thinning (*p* < 1%). In the GCC significance map, the colors represent the deviation from the normative database: green indicates normal thickness, yellow indicates borderline thinning (*p* < 5%), and red indicates significant thinning (*p* < 1%). The glaucomatous eye demonstrates higher Vertical Asymmetry Score (V-Asy) and Quadrantal Asymmetry Score (Q-Asy) compared with the fellow normal eye (normal eye: V-Asy = 5.45, Q-Asy = 4.62; glaucomatous eye: V-Asy = 10.66, Q-Asy = 7.61). This example illustrates how the proposed asymmetry metrics can visualize and quantify macular asymmetry associated with glaucomatous structural change in a unilateral case.

Table 2 summarizes the macular OCT measurements. The mean inner retinal layer thickness was significantly thinner in glaucomatous eyes compared with normal eyes. The superior–inferior thickness difference was also greater in glaucomatous eyes. Both the Vertical Asymmetry Score and the Quadrantal Asymmetry Score were significantly higher in the glaucoma group than in the normal group. A representative case is shown in Figure 3, illustrating a 48-year-old woman with unilateral glaucoma. The glaucomatous eye exhibited markedly increased Vertical Asymmetry (10.66) and Quadrantal Asymmetry (7.61) compared with the fellow normal eye (Vertical Asymmetry 5.45; Quadrantal Asymmetry 4.62), demonstrating the ability of these metrics to capture macular asymmetry associated with glaucomatous structural change.

### 3.3. Diagnostic Performance

**Figure 4:**
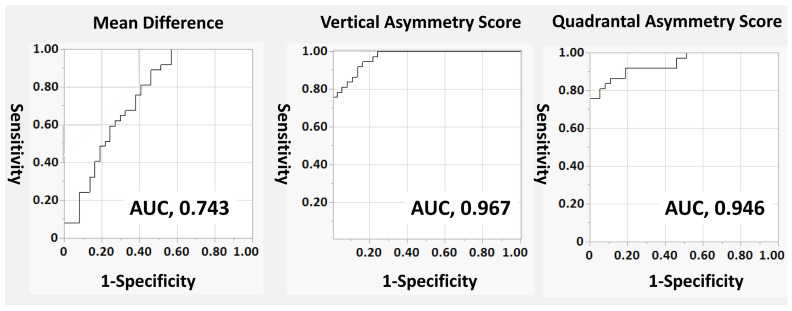
Receiver operating characteristic (ROC) curves demonstrating the diagnostic performance of three macular OCT parameters for detecting glaucoma. The ROC curves compare the mean inner retinal thickness difference (Mean Difference), the Vertical Asymmetry Score, and the Quadrantal Asymmetry Score. The area under the curve (AUC) values were 0.743 for the Mean Difference, 0.967 for the Vertical Asymmetry Score, and 0.946 for the Quadrantal Asymmetry Score. Both the Vertical Asymmetry Score and Quadrantal Asymmetry Score showed markedly superior diagnostic accuracy compared with the conventional superior–inferior thickness difference measure.

Table 3 and Figure 4 present the diagnostic accuracy of each parameter for glaucoma. The area under the ROC curve (AUC) was 0.743 (95% CI, 0.627–0.859) for the mean inner retinal thickness difference, 0.967 (95% CI, 0.937–0.999) for the Vertical Asymmetry Score, and 0.946 (95% CI, 0.898–0.994) for the Quadrantal Asymmetry Score. Both the Vertical Asymmetry Score and the Quadrantal Asymmetry Score demonstrated significantly greater diagnostic performance than the conventional superior–inferior thickness difference.

## 4. Discussion

In this study, we demonstrated the ability to detect glaucomatous eyes more sensitively than conventional imaging by utilizing the difference in macular superior and inferior hemiretina thickness, emphasized through a linearization program for the temporal raphe sign. This was made possible by employing a linearization program to meticulously analyze the differences in thickness between the superior and inferior hemiretina in the macular region.

Recent progress in OCT technology has been pivotal in advancing our understanding of glaucoma, particularly in its early stages. A key discovery has been the differentiation in thickness of the inner retinal layers located temporally to the macula. This difference, observed across the horizontal meridian, was first noted by Kim et al. in 2015 [8]. They described this phenomenon as ‘Hemifield Difference across Horizontal Raphe’ and underscored its diagnostic value in glaucoma detection. Moreover, these researchers also pointed out that the temporal raphe sign, a specific change observed in glaucomatous eyes, is instrumental in distinguishing glaucoma from other types of optic neuropathies [9]. Our study’s results align with these previous findings, providing further evidence of the sign’s diagnostic significance.

One challenge in the study of glaucoma has been the anatomical variations between individuals, particularly concerning the relative positions of the optic nerve head and the macula. It has been reported, for instance, that these two critical eye components are not aligned at the same height and that there is considerable individual variability in the angle formed between them. Hood and colleagues have contributed to this understanding by reporting that, on average, the fovea is situated approximately 8 degrees below the optic nerve head [15]. However, they also highlighted the existence of substantial individual differences in this positioning. The fovea is generally located approximately 6 to 8 vertically below the optic disc in healthy individuals with wide interindividual variation [16,17,18].

The asymmetry in the trajectory of nerve fibers extending from the optic nerve head to the macula is an important aspect to consider. This is especially true when examining the thickness of the peripapillary retinal nerve fiber layer (cpRNFL), a quantification technique that has been utilized since the time of Time-Domain OCT (TD-OCT). The Garway-Heath map’s asymmetric nature is also linked to its evaluation methodology, which centers around the optic nerve head. Despite the advancements in OCT technology, current commercially available OCT models with built-in programs can only measure the thickness of the inner retinal layers in a superior and inferior orientation. However, none of these models are equipped to detect asymmetry scores. Our study addresses this gap by developing an automatic program that linearizes the optic nerve head and the temporal raphe, thereby enhancing the ability to detect glaucoma. Typically, the optic nerve head information is incorporated in the assessment, leading to an expansion of the superior retina and a contraction of the inferior retina. After this adjustment, the program calculates the difference in inner retinal layer thickness at each symmetric point, as well as the average value and standard deviation of these log-ratios. The significance of this approach lies in its ability to capture local variability, as the variance in thickness differences increases with localized disparities. This is particularly relevant in early-stage glaucoma, where local thinning of the nerve fiber layer thickness is a characteristic structural change.

It is widely acknowledged that the inner retinal layer undergoes thinning as glaucoma progresses. However, there is also natural individual variation in the thickness of this layer in healthy eyes. Distinguishing between thinning due to early glaucomatous structural changes and mere individual differences poses a significant challenge when relying solely on average measurements of inner retinal layer thickness. Glaucoma is a leading cause of blindness in developed countries, and the thickness of the macular retina is closely associated with the fixation point, directly impacting the quality of vision (QOV). Accordingly, objective and standardized quantification of macular inner retinal layer patterns is clinically relevant. In this study, our fully automated linearization program enabled consistent geometric alignment along the disc–fovea axis and temporal raphe, and the proposed asymmetry scores provided an objective description of macular asymmetry in a fellow-eye–controlled unilateral dataset. These metrics may complement conventional thickness-based evaluation, particularly when unilateral or asymmetric glaucomatous involvement is suspected. However, the applicability to eyes with high myopia or other concomitant fundus pathologies was not evaluated in the present cohort (these eyes were excluded) and requires dedicated validation in future studies.

We acknowledge several limitations in our study. First, the current study design employed a within-subject comparison between glaucoma eyes and fellow normal eyes. This approach was chosen to minimize inter-subject anatomical variability and purely evaluate the technical precision of the algorithm. While this design serves as a robust “proof of concept,” the high diagnostic performance (AUCs) reported here may not be directly generalizable to real-world screening contexts or populations with bilateral disease, where inter-subject variability plays a larger role. Second, the Quadrantal Asymmetry Score utilizes data from the fellow eye. While effective for detecting pathological asymmetry, this metric depends on the fellow eye being healthy. Its utility may be limited in cases of bilateral symmetric glaucoma or when the fellow eye has co-morbidities. Third, the linearization program involves geometric transformation. Although it effectively corrected the disc-fovea angle, further research is necessary to confirm its structural accuracy. Fourth, our study was limited to Asian (Japanese) subjects. As average retinal thickness varies by race, reference values for symmetry scores may differ in other populations. Fifth, although we excluded eyes with clinically significant cataracts, mild lens status differences could affect global thickness measurements; however, our ratio-based scores are theoretically less susceptible to generalized signal attenuation. Finally, regarding the applicability to other conditions such as high myopia mentioned earlier, this remains speculative as these eyes were excluded from the current study. Future large-scale studies are warranted to determine robust cut-offs and validate the method in diverse populations including those with high myopia.

In conclusion, the linearization correction program and novel asymmetry scores demonstrated potential utility for glaucoma detection compared to simple thickness difference metrics. This approach, which focuses on the temporal raphe sign, shows promise in detecting glaucomatous structural changes. Further validation in diverse cohorts is warranted.

## Figures and Tables

**Table 1:** The Demographic and Clinical Characteristics of the Study Participants.

	Healthy	Glaucoma	*p* Value
**number of patients**	37		
**Age (years)**	68 (50, 76)		
**Sex (male/female)**	14/23		
**Axial length (mm)**	24.3 (23.1, 26.7)	24.4 (23.2, 26.8)	0.798
**Central corneal thickness (μm)**	521 ± 30	522 ± 36	0.980
**Visual acuity (LogMAR)**	−0.08 (−0.08, −0.08)	−0.08 (−0.08, 0.03)	0.186
**Intraocular pressure (mmHg)**	13.6 ± 3.5	13.1 ± 4.6	0.636
**HFA 24-2 MD (dB)**	0.2 (−2.0, 0.7)	−4.7 (−11.7, −2.1)	<0.001

Abbreviations: HFA = Humphrey Field Analyzer; MD = Mean deviation; LogMAR = logarithm of the minimum angle of resolution. For normally distributed variables, results are shown as mean ± standard deviation; for non–normally distributed variables, results are shown as median (interquartile range). Data expressed as mean ± standard deviation were compared with paired *t* test. Data expressed as the median (interquartile range) were compared using the nonparametric Wilcoxon signed rank test.

**Table 2:** Macular OCT Parameters in Normal and Glaucomatous Eyes.

Variables	Healthy	Glaucoma	*p* Value
**Inner Retinal Thickness**			
Average (µm)	93.4 (90.1, 98.5)	80.3 (77.3, 85.0)	<0.001
Superior (µm)	93.8 (90.2, 97.5)	81.6 (76.6, 88.9)	<0.001
Inferior (µm)	93.5 (90.1, 98.4)	78.4 (73.1, 88.6)	<0.001
Mean Difference (µm)	2.66 (1.65, 5.17)	6.94 (2.80, 12.54)	<0.001
**Asymmetry Score**			
Vertical Asymmetry Score	6.80 (6.15, 7.25)	9.69 (9.16, 11.58)	<0.001
Quadrantal Asymmetry Score	6.35 (5.94, 7.19)	8.47 (8.11, 9.63)	<0.001

Results are shown as median (interquartile range).

**Table 3:** Diagnostic Performance of Macular OCT Parameters for Detecting Glaucoma.

Variables	AUC (95%CI)	*p* Value *	*p* Value ^†^
Mean Difference	0.743 (0.627–0.859)	Reference	
Vertical Asymmetry Score	0.967 (0.937–0.999)	<0.001	Reference
Quadrantal Asymmetry Score	0.946 (0.898–0.994)	<0.001	0.238

* *p* value when the reference value is the AUC of the mean difference; ^†^ *p* value when the reference value is the AUC of the vertical asymmetry score.

## Data Availability

The datasets generated and/or analyzed during the current study are available from the corresponding author upon reasonable request.
